# Continuities and discontinuities in the cultural evolution of global consciousness

**DOI:** 10.1098/rstb.2022.0263

**Published:** 2024-01-01

**Authors:** Robert Jiqi Zhang, James H. Liu, Michelle Lee, Mei-hua Lin, Tian Xie, Sylvia Xiaohua Chen, Angela K.-y. Leung, I-Ching Lee, Darrin Hodgetts, Evan A. Valdes, Sarah Y. Choi

**Affiliations:** ^1^ School of Psychology, Nanjing Normal University, People's Republic of China; ^2^ School of Psychology, Massey University, 0745, New Zealand; ^3^ Department of Psychology, Sunway University, Selangor, Malaysia; ^4^ Department of Psychology, Philosophy School, Wuhan University, People's Republic of China; ^5^ Department of Applied Social Sciences, Hong Kong Polytechnic University, People's Republic of China; ^6^ School of Social Sciences, Management University, Singapore; ^7^ Department of Psychology, National Taiwan University, Taiwan

**Keywords:** identification with all humanity, cosmopolitanism, social learning, death, global consciousness, cultural evolution

## Abstract

Global consciousness (GC), encompassing cosmopolitan orientation, global orientations (i.e. openness to multicultural experiences) and identification with all humanity, is a relatively stable individual difference that is strongly associated with pro-environmental attitudes and behaviours, less ingroup favouritism and prejudice, and greater pandemic prevention safety behaviours. Little is known about how it is socialized in everyday life. Using stratified samples from six societies, socializing institution factors correlating positively with GC were education, white collar work (and its higher income) and religiosity. However, GC also decreased with increasing age, contradicting a ‘wisdom of elders’ transmission of social learning, and not replicating typical findings that general prosociality increases with age. Longitudinal findings were that empathy-building, network-enhancing elements like getting married or welcoming a new infant, increased GC the most across a three-month interval. Instrumental gains like receiving a promotion (or getting a better job) also showed positive effects. Less intuitively, death of a close-other enhanced rather than reduced GC. Perhaps this was achieved through the ritualized management of meaning where a sense of the smallness of self is associated with growth of empathy for the human condition, as a more discontinuous or opportunistic form of culture-based learning.

This article is part of the theme issue ‘Evolution and sustainability: gathering the strands for an Anthropocene synthesis’.

## 1. Introduction

The past century has witnessed such unprecedented, multi-facetted and pervasive human-driven planetary change that a working group of the International Geological Society recommended that ‘the Anthropocene be established [as] a new geological epoch, with a start date in the mid-twentieth century’ [1]. The signal event of the Anthropocene is climate change manufactured by human activity, with average planetary temperature projected to rise by 2.6°C by the end of the century [2]. The environmental unsustainability of the current trajectory means that theories of cultural evolution, centring around *continuities* of intergenerational social learning [3], could usefully be augmented by a political psychology that identifies moments of potential and actual *discontinuity* [4] that could be leveraged for social, institutional and personal change. We discuss global consciousness (GC) [5] as an individual difference especially adaptive for the human species to survive (and thrive) in the Anthropocene. GC and related concepts are strongly related to pro-environmental attitudes and behaviours [6–9] and cross-national cooperation [10]. We review what macro-features of society induce GC to grow (or decline), but argue further that discontinuity in individual and collective lives might open up a window for reconceptualizing the forms of experiential meaning that give rise to GC.

Evolutionarily, GC is rooted in biologically heritable characteristics like the personality trait of agreeableness, and the ability to feel empathy, which facilitate prosocial behaviour [11]. Beyond prosociality at the interpersonal level, evolved abilities to be tolerant of out-group members allowed the formation of profitable cooperative relationships with non-kin, as in geographically extensive trading networks documented in prehistory [12]. Furthermore, prosociality can also involve self-sacrifice on behalf of the collective or for group survival, tendencies that can be explained by cultural group selection [13,14]. The capacity to submit to group norms enforcing cooperation [14], including religious beliefs in moralizing gods, may have enabled the formation of large-scale human collectives [15] with a competitive advantage over groups more prone to engaging in intergroup conflict. However, the same cultural features responsible for peace-making can also be used for making war [12], so it is an open question to what extent the capacity for prosocial behaviour on behalf of a collective could be so flexible and extensible to encompass GC. Cultural group selection is especially influential in evolutionary environments involving intergroup conflict [15,16], and in-group favouritism is highly consistent across national boundaries today [17].

In the words of Charles Darwin, as cited in Richards [13], ‘although a high standard of morality gives but a slight or no advantage to each individual man and his children over the other men of the same tribe, yet that an advancement in the standard of morality and an increase in the number of well-endowed men will certainly give an immense advantage of one tribe over another’ (p. 145).

It does take a leap of faith to conceive of this tribe becoming global and acquiring values calibrated to be compatible with global institutions, as in the most famous articulation of GC put forward by enlightenment era philosopher Immanuel Kant [18]. His cosmopolitan vision has a top-down institutional structure consisting of a federation of states, complemented by bottom-up growth of cosmopolitan individuals who fulfil roles for such a federation to function peaceably and well. Given the dominance of national and local identities over the global identity [19], and the relative power of states over a federation of states internationally, our focus here is on the bottom-up side of Kant's vision: what lived experiences, encompassing both continuity and discontinuity, cultivate GC for individuals [5]?

This work builds on efforts to develop quantitative measures of what McFarland *et al*. [20] ecumenically termed ‘Global Human Identification and Citizenship’. These encompass a wide range of measures that arguably fit with Liu and McDonald's [5] definition of GC as ‘a knowledge of both the interconnectedness and difference of humankind, and a will to take moral actions in a reflexive manner on its behalf’ (p. 310). GC is cosmopolitan [9], incorporating global prosociality (a willingness to help others regardless of whether they share an ingroup with the self) and respect for and willingness to preserve cultural diversity. It is fundamentally open towards acquiring knowledge about other cultures, other peoples and other lifeways. Conversely, GC is not defensive about maintaining ethnic purity or anxious/fearful about interacting with others different from oneself [21]. GC includes a feeling of ‘we-ness’, or identification with all humanity, including both bonding with and concern for all humanity [19,22]. Measured as a composite, GC has good test–retest reliability and predicts cooperation across national boundaries on a range of behavioural indicators [10], rather than being narrowly defined by a particular theoretical orientation or literature. Most relevant to the current research, it is a person-centred measure that allows researchers to track personal change longitudinally, as a function of life experiences.

McFarland *et al*.'s [20] review observed that regardless of how you measure global human identification and citizenship, its correlations with stable individual differences are theoretically intuitive and consistent, but small in magnitude. Among the constituent elements of GC, we know that cosmopolitanism is correlated with the personality traits of agreeableness and openness [7], global orientations is linked to extraversion, openness to experience, and intellect [21], and global identification [19] is positively related to dispositional empathy, openness to experience, and the values of universalism, care, and justice. Conversely, all of them have negative correlations to prejudicial individual differences like social dominance orientation and right-wing authoritarianism. The literature's focus on psychological correlates hence shows that genetically inherited dispositions contribute a kernel of potential, but more fully fledged GC grows or declines over the course of an individual's lifespan through life experiences and social learning. Very little research has examined this from the perspective of everyday life.

Rather, research using multi-national samples has shown that individuals high in GC are more cooperative, and hence suffer greater losses when placed in a competitive situation (a prisoner's dilemma) involving real money being exchanged within and across national borders [10]. Balanced against this lack of evolutionary survivability in a dyadic situation where selfishness results in a better payoff than cooperativeness, other research shows that GC [23] and its elements (identification with all humanity [24] and cosmopolitan orientation [25]) predict adopting more safety measures against COVID-19, better collaboration to contain the pandemic and greater willingness to be vaccinated. Therefore, GC is likely to thrive in situations where cooperation provides survival benefits to the individual as part of a collective, and to suffer in situations (e.g. social dilemmas) where cooperativeness is pitted against selfishness at the individual level. GC is therefore, like other cooperative orientations, vulnerable to decline when faced by external threat [26] at either the group or the individual level [16]. Like trust, it thrives in situations where there is a rule of law that allows mutual gains to be made from cooperative economic exchanges and suffers in situations where there is gain to be made from cheating or exploiting others [27]. Thus, we have a decent understanding of macro-level features of society that can facilitate or impede the growth of GC.

However, we only have a bare outline of how GC is socialized. Personal belief in the major religions has small positive correlations with cosmopolitanism [28]. This is consistent with Norenzayan *et al*.'s [15] culture evolution thesis that big religions enhance prosocial behaviour and allow societies to grow larger in scale. Reysen and colleagues [29,30] found that structured life experiences (taking university classes, consuming news) that increase knowledge about the world increase global citizenship identification. Similarly, GC can be taught in schools, through global citizenship education [31]. This suggests that some aspects of GC can be socially learned through processes involving continuity, like the transmission of religion or official schooling [3]. However, other aspects of GC involve less traditional and more experiential/opportunistic sources of learning. Römpke *et al*. [32] found that international contacts increased identification with all humanity; Sparkman & Eidelman [33] found the same for multicultural experiences. Such forms of selective and opportunistic experience might be one reason why McFarland *et al*. [34] found no correlations between recall of how one was raised as a child (including punitiveness by parents, a supposed hallmark for acquiring authoritarianism) and identification with all humanity as an adult. These mixed results inform the current research.

### (a) Learning global consciousness (GC)

We examine how learning affects GC, a) correlationally, through one's position in societal structures that reproduce social learning over the lifespan, and b) dynamically, through longitudinal analysis of the occurrences of major life events that may result in disruptions and discontinuities in the immediate short term.

Because the literature on GC is young, we frame this analysis using correlational research on lifespan development, which converges on the finding that in modern societies, prosociality increases with age. Bailey *et al*.'s [35] review highlights adults typically becoming more other-oriented as they age because of the social roles they assume: ‘ageing stimulates an accommodative process of disengagement from individualistic future-oriented goals (power, achievement, and competence) and an orientation toward ego-transcending strivings' (p. 2).

However, general prosociality might not relate to GC in a straightforward way. Older adults are typically oriented towards significant others, with whom the older person has formed a meaningful and enduring attachment. A study of over 67 000 individuals across 67 countries [36] found that donations overall—and specifically to a national charity—increased with age, but donations to an international charity decreased with age. GC would appear to require specific socialization experiences rather than age-based maturation, as previous research has shown it to be positively correlated to religiosity [28], higher education [20] and socio-economic status [7].

Matsumoto *et al*.'s [37] path model shows older adults being more prosocial in making resource allocations across a range of experimental games (including the prisoner's dilemma and dictator games) because of dissatisfaction with use of a non-cooperative strategy to exploit others, coupled with lower belief that manipulating others is required for success in life. These two variables mediated the relationship between older age and more prosocial allocation decisions, thus highlighting that prosociality could be learned from specific life experiences involving relationships loss. Matsumoto *et al*. [37] speculate that ‘unilateral defection is not an attractive option because they intuitively associate it with the long-term outcome of mutual defection’ (p. 13). According to this interpretation, older adults have learned that unilaterally cheating others leads to relationship dissolution, and so they tend to do it less.

In summary, we examine whether GC increases as a function of age, providing a test of whether it functions as a form of ‘wisdom of elders' [38]. We also extend previous research on its structural correlates by adding income irregularity and occupation [39] to previous research on structural correlates that emphasize continuity in social learning [3].

Little is known of the dynamics of GC changing as a function of specific life events. At first intuition, one might anticipate that GC increases with positive events in one's life, just like the more established finding on the other end of life experience, that there is a one-to-one mapping between negative life experiences and increased depression [40] and lesser subjective well-being [41]. It stands to reason that positive events like marriage, the birth of a child or promotion at work might be associated with the growth of GC, through the expansion of empathy, the enlarging of social networks and the rewarding of cooperative behaviour in one's social networks.

However, there is a dearth of theory that seeks to explain what might happen to GC when negative (but unavoidable) events like illness, a death in the family or economic downturns in life occur. Only in the domain of death is there a clear theory-based hypothesis, from terror management theory (TMT) [42]: mortality salience is perceived as a source of threat that activates a defensive worldview (including outgroup stereotypes and prejudice) that should, in turn, decrease GC. However, most of the research on TMT uses experiments with American undergraduates, and mortality salience effects are not uniform across other situations and demographics [43].

By contrast, Zhang's [44] research on dark tourism (sites of death and destruction) suggests a more complicated dialectic between positive and negative life experiences in fostering GC; summarizing the literature, she writes ‘A lack of faith or religion potentially creates anxiety, vulnerability, and isolation in the face of death and dying… Consuming dark tourism may reflect this state of anxiety in individuals and expresses their pursuit for ontological security in contemporary society’. This anxiety motivates a search for existential authenticity; it may stimulate awe, and awe and profundity can make the self aware of how small it is in the greater scheme of things [45], but also interconnected: serving as an affirmation of life across generational time, expanding the boundary of who one is, perhaps through ritual affirmation of ancestral ties [46]. No research we are aware of has attempted to track the impact of a death in the family longitudinally on the growth or decline of GC. This is an experience of discontinuity rather than continuity, but that is simultaneously a part of life scripts in every culture.

Hence, this research reports the effects of prototypical contemporary life experiences on GC, including positive (getting married, birth of a child, promotion at work, education achievement), negative (death in the family, loss of work, financial setback) and neutral events (relocation, retirement).

## 2. Method

### (a) Participants

Data came from a 2-wave online survey with samples from China (Mainland), Hong Kong, Malaysia, Singapore, Taiwan and the United States. There was a 3-month interval between waves: wave 1 data were collected from July 2 to July 16, 2020, involving 6138 participants; wave 2 data were collected from October 5 to November 8, 2020, with 1449 participants retained. Participants were recruited through Toluna, a global polling company. The selection of participants was stratified by age, gender and socio-economic status (see detailed sample demographics in tables 1 and 2).

**Table 1.** Demographics based on wave 1 data (*N* = 6138). SES, socioeconomic status.

	Mainland China	Hong Kong	Malaysia	Singapore	Taiwan	USA
N _wave1_	1015	1000	998	998	998	1123
N _wave2_	220	337	188	205	314	185
gender						
female	524	581	487	420	520	602
male	491	419	511	578	478	521
age	37.3 (10.3)	40.8 (10.3)	37.4 (10.2)	41.6 (11.3)	39.4 (10.2)	54.0 (14.9)
occupation						
owner	9	13	93	56	26	62
executive	342	220	221	431	112	192
professional	214	146	211	230	199	153
technician	163	81	39	26	146	25
clerical	141	316	117	74	161	60
sales/service	38	86	58	50	122	56
agricultural	2	3	11	1	2	7
trade	9	15	15	1	9	9
unskilled	14	22	11	1	37	3
armed forces	3	4	6	5	11	1
homemaker	7	36	51	28	59	93
unemployed	15	26	57	34	50	89
student	18	6	17	8	7	8
other	41	26	89	53	57	365
income (median)	15 649.45	38 610.04	8674.70	44 776.12	17 940.44	50 000.00
income ppp (median)	3743.89	6360.80	5525.29	53 304.90	1344.86	50 000.00
income deciles	5.49 (2.79)	5.55 (2.89)	5.47 (2.88)	5.52 (2.86)	5.50 (2.88)	5.50 (2.87)
income						
income regularity	5.2 (1.2)	4.9 (1.5)	4.7 (1.6)	5.4 (1.6)	4.6 (1.6)	5.6 (1.6)
subjective SES	5.4 (1.6)	4.7 (1.7)	5.7 (1.8)	6.1 (1.9)	5.0 (1.7)	5.9 (2.1)
education	4.8 (0.7)	4.3 (1.1)	4.4 (1.1)	4.8 (1.0)	4.8 (1.0)	4.6 (1.0)
personal religiosity	1.6 (0.8)	1.9 (0.9)	3.3 (0.9)	2.6 (1.0)	2.2 (0.9)	2.6 (1.1)
organizational religiosity	2.0 (0.7)	2.1 (0.8)	3.1 (1.0)	2.2 (1.0)	2.2 (0.8)	2.2 (1.1)

*Note:* s.d.s are contained within parentheses. Income ppp was calculated using Purchasing Power Parity (PPP) conversions. Medians instead of means are presented for income and income ppp.

**Table 2.** Religion descriptives based on wave 1 data (*N* = 6138). GC, global consciousness.

religion	*N*	high GC %	income regularity	subjective SES	personal religiosity	organizational religiosity
Buddhist	934	35.0%	4.8 (1.5)	5.5 (1.7)	2.5 (0.8)	2.2 (0.9)
Christian	1644	42.8%	5.4 (1.6)	5.9 (2.1)	3.0 (0.9)	2.6 (1.0)
Muslim	688	42.2%	4.7 (1.6)	5.9 (1.9)	3.8 (0.6)	3.5 (0.8)
Taoist	294	39.8%	5.0 (1.6)	5.3 (1.8)	2.4 (0.8)	2.2 (0.7)
not religious	2015	33.8%	5.1 (1.5)	5.1 (1.7)	1.3 (0.6)	1.8 (0.7)
other (e.g. Hindu, Agnostic, folk religion)	557	40.2%	5.0 (1.6)	5.2 (1.9)	2.2 (1.0)	2.1 (0.9)

*Note:* s.d. s are contained within parentheses. Not religious people are more prevalent than religious people in China and Hong Kong.

### (b) Measures

GC was measured in both wave 1 and wave 2 by 3 relevant scales: cosmopolitan orientation (with subfactors of global prosociality, openness to culture and respect for cultural diversity [9]), global orientations (with 2 subfactors, multicultural acquisition and ethnic protection [21]) and identification with all humanity (with 2 subfactors, bonding and concern; see [19,47,48]). Following Liu *et al*. [10], we used latent profile analysis to bind the 7 subscales together to provide a holistic latent profile (i.e. high, medium and low) of global consciousness.

### (c) Demographic variables (all measured in wave 1)

Income was measured by asking ‘how much money (in the local currency) do you earn annually’? The unit of currency was *Yuan* for mainland China, *Hong Kong dollar* for Hong Kong, *Ringgit* for Malaysia, *Singapore dollar* for Singapore, *Taiwan dollar* for Taiwan, and *US dollars* for the USA.

Income regularity was measured by asking participants how regular or irregular their income is? The answers ranged from 1 = *very irregular* to 7 = *very regular*.

Occupation was measured by asking participants to describe their current occupation with the following options: 1 = business owner, 2 = manager/executive, 3 = professional, 4 = technician, 5 = clerical support workers, 6 = service and sales worker, 7 = skilled agricultural, forestry or fishery worker, 8 = craft and related trades worker, 9 = elementary occupations, 10 = armed forces, 11 = homemaker, 12 = unemployed, 13 = student, 14 = other. Answers were recoded into 1 = white collar work (including options 1, 2 and 3) and 0 = other work.

Education was measured by asking the highest level of education that the participant has completed. The answers included: 1 = *elementary school*, 2 = *middle school*, 3 = *high school*, 4 = *some college*, 5 = *bachelor's degree at university*, 6 = *graduate degree or higher*.

Subjective socio-economic status was measured by asking ‘on a scale of 1 to 10, with 10 being people who are the most well off in society and 1 being the people who are least well off, where would you describe your position?’. The answers ranged from 1 = *least well off* to 10 = *most well off*.

Personal and organizational religiosity was measured by 2 single-item questions. Personal religiosity was measured by asking ‘how important is religion to your life’ (from 1 = *not important* to 4 = *extremely important*) and organizational religiosity was measured by asking ‘what part do you think religion should play in governing a country’ (from 1 = *no part at all* to 4 = *an extremely important part*).

### (d) Life experience changes (wave 2)

Life experience changes were measured in wave 2 by 15 items, including (1) moved to a new location; (2) completed a degree or educational certificate; (3) being promoted to a better position; (4) started a new job or found a better job; (5) lost my job or am feeling insecure about my job; (6) retired or am preparing for retirement soon; (7) had a major financial setback; (8) got married or started a new romantic relationship; (9) gained a new family member (e.g. new child); (10) broke up, got divorced or having serious relationship troubles; (11) someone close to you died; (12) someone close to you became ill and requires care; (13) the last of my children has left home (empty nest); (14) trying some new activities; and (15) other. Participants were asked to indicate whether or not a particular event happened in the past few months (1 = *did happen* and 0 = *did not happen*). These items were selected through a literature review of the life experiences literature (e.g. [41,49,50]).

## 3. Results

### (a) Relationship between global consciousness (GC) and demographics

We employed latent profile analysis to incorporate the 3 scales and 7 subscales relevant to GC. As a 3-profile solution (high GC, medium GC and low GC) was selected as the optimal model in a previous study using the same dataset (Liu *et al*., under review), we used this solution as the base to examine the association between GC profiles and key demographic variables. A three-step approach was used for this purpose [51]. Cohen's [52] *ω*^2^ was calculated to indicate the strength of these associations: 0.1, 0.3 and 0.5 indicate small, medium and large effect sizes, respectively.

Small differences were observed for age, in that people with high GC were youngest, followed by medium GC, and low GC was the oldest profile. No difference was found for gender.

Small to medium differences were observed for indicators of status, including income, income regularity, occupation, education and self-judged socioeconomic status. Consistently across all indicators, people in the high GC profile had significantly higher status than people with medium GC and low GC, and people in the medium GC profile had significantly higher status than people with low GC.

Small to medium differences were also observed for two indicators of religiosity. Specifically, people with high GC had significantly higher levels of personal and organizational religiosity than people in medium GC and low GC, but those with medium GC and low GC did not have significant differences in religiosity (table 3).

**Table 3.** Differences between GC in key demographics based on the LPA results with wave 1 data. ***, *p* < 0.001.

	high GC (A)	medium GC (B)	low GC (C)	approximate *χ*^2^	*ω*^2^
age	40.3^B, C^	42.3^A, C^	45.3^A, B^	98.359***	0.127
gender (male %)	50.2%	47.1%	51.2%	4.969	0.028
income (deciles)	6.0^B, C^	5.4^A, C^	4.6^A, B^	168.434***	0.176
income regularity	5.4^B, C^	5.0^A, C^	4.6^A, B^	170.542***	0.167
occupation (white collar %)	56.9%^B, C^	44.9%^A, C^	32.9%^A, B^	147.821***	0.155
education	4.8^B, C^	4.5^A, C^	4.2^A, B^	255.766***	0.204
subjective SES	6.0^B, C^	5.3^A, C^	4.8^A, B^	300.986***	0.222
personal religiosity	2.6^B, C^	2.2^A^	2.1^A^	199.285***	0.180
organizational religiosity	2.5^B, C^	2.2^A^	2.2^A^	92.258***	0.123

*Note:* Effect size was calculated by *ω*^2^ = (*χ*^2^/N)^1/2^. Superscripts indicate profiles that are significantly different at *p* < 0.05.

**Figure 1.**
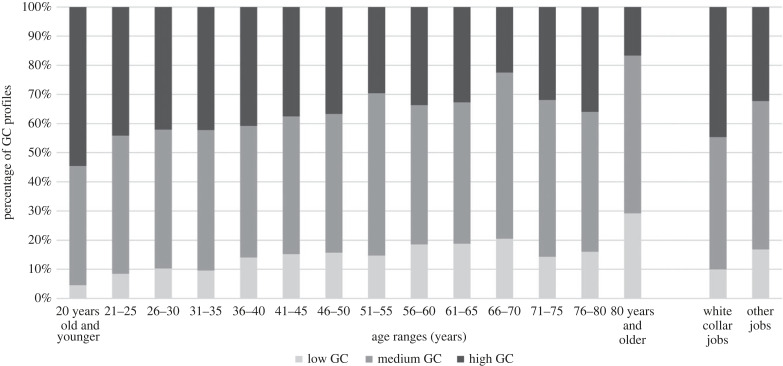
Proportions of GC profiles in different age groups and occupations.

To further inspect the potential interaction effect of age and occupation, we conducted logistic regression in which the main effects of age (both linear and quadratic effects) and occupation, and their interactions, were included as predictors and GC profiles (recoded as 1 = high GC and 0 = medium or low GC) were the dependent variable. Results suggested that both age and occupation were associated with the odds of being in the high GC profile. For every one unit increase in age (one year older), the odds of being high GC decreased by 0.985 (a tiny effect); whereas the odds ratio of being in the high GC profile for people with white-collar work was 1.737 times higher than for people with blue collar and other jobs (figure 1).

However, no interaction effect between age and occupation was found. This suggests GC is new rather than traditional knowledge, that does not follow a ‘wisdom of the elders' social learning path of culture-level transmission, but rather is associated with age-specific cohort effects where knowledge is acquired through socialization into an occupation [38]^1^.

### (b) Relationship between global consciousness (GC) and life experiences

To examine the impact of life event changes on GC, a set of latent transition analyses (LTA) [54] were estimated using the robust likelihood estimator (MLP) in Mplus 7.31. Only those participants who responded in both wave 1 and wave 2 surveys (*N* = 1449) were included for the following analyses^2^. We constructed the LTA models with 3 profiles and include longitudinal invariance constraints.

To assess the prevalence of latent profile and transition probabilities, we first estimated an LTA model without covariates (table 4). Results suggested that over the 3-month between-wave interval, the proportion of high GC increased from 24.7% to 27.5%, medium GC decreased from 49.7% to 47.5% and low GC dropped from 25.6% to 25.0%. All 3 GC profiles were relatively stable across waves, with 93.2%, 85.3%, and 86.5% retaining in the same profile for high, medium and low GC. However, overall advance rate, weighted by the proportion of each profile, was 10.8%, indicating that roughly 1 in 10 participants advanced from a lower GC in wave 1 to a higher GC in wave 2 (for example, from low GC to medium/high GC, or from medium to high GC).

**Table 4.** Prevalence of 3 global consciousness profiles and transition probabilities based on latent transition analysis without covariates.

	high GC	medium GC	low GC
	*proportions for latent profiles*		
wave 1	0.247	0.497	0.256
wave 2	0.275	0.475	0.250
	*transition probabilities (rows for wave 1 and columns for wave 2)*		
high GC	0.932	0.065	0.003
medium GC	0.093	0.853	0.054
low GC	0.017	0.120	0.863

**Figure 2.**
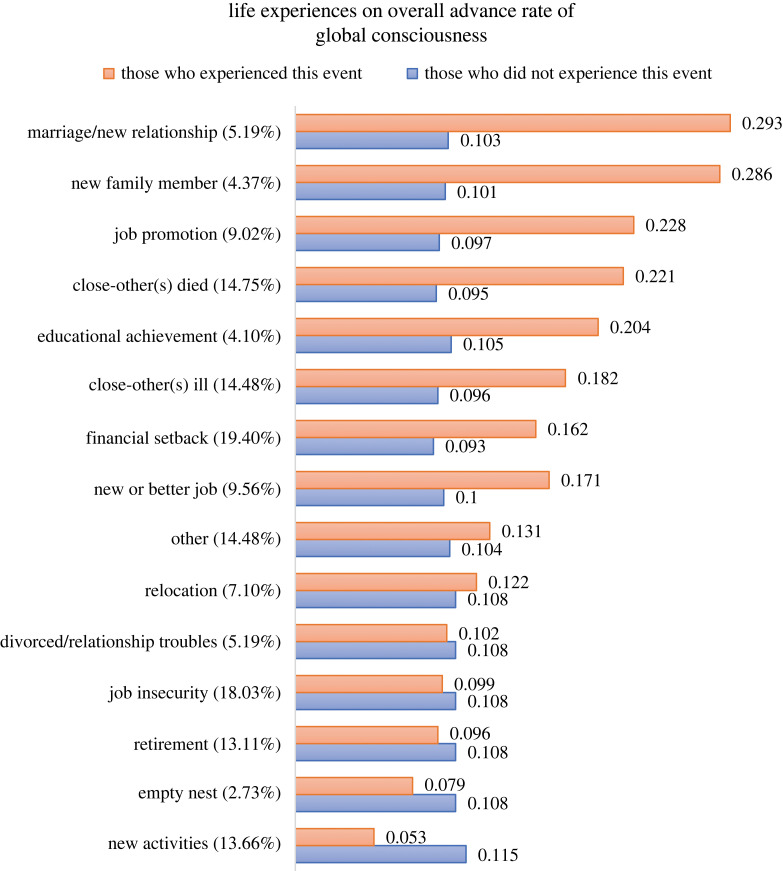
Life experiences on overall advance rate of global consciousness. *Note:* The overall advance rates (the numbers next to the bars) are calculated by averaging the advance rates of low GC profile and medium GC profile, weighted by the probability of the correspondent profile at wave 1. The numbers in the parentheses after the event are the proportions of participants who experienced that event in the past few months. Events are sorted by ratio of experiencing a particular event to not experiencing that event (from the highest to the lowest).

Then, we estimated 15 LTA models with covariates, where a life event was used as the covariate for each model. With those analyses, we were able to estimate probabilities of transitioning from a GC profile in wave 1 to another one in wave 2, conditioned on life events (all the detailed results are presented in electronic supplementary material, table S33, including the odds of transitioning from a higher level to a lower level of GC^3^). Therefore, we could further calculate the advance rates for those who experienced a particular life event versus those who did not experience it (figure 2). Among all the events in our measure that had a positive impact on GC (i.e. the advance rate for those who experienced a particular event was higher than those who did not experience it), marriage/start of a new relationship (2.8) and having a new family member (2.8) had the strongest impact, with the chance of advancing to a higher GC being 2.8 times higher for those who experienced it compared to those who did not. Other events that positively affected GC include job promotion (2.4), the death of someone close to you (2.3), educational achievement (1.9), illness of someone close to you (1.9), financial setback (1.7), having a new or better job (1.7). Other unspecific events (1.3), and relocation (1.1) were fairly close to having no net effect. Events like becoming an empty nester (0.7), retirement (0.9), job insecurity (0.9) and divorce/relationship troubles (0.9) had slight negative impacts on GC. Surprisingly, new activities had the most detrimental impact on GC (0.5), reducing the advancement chance of those who experienced them to half of those who did not. Inspection of open-ended answers revealed participants mentioning new activities relating to COVID-19 pandemic, such as working at home and wearing a mask, which may explain its negative impact on GC.

## 4. Discussion

As humanity moves into the Anthropocene epoch [1,55–57], it is apparent that ‘industry as usual’ is unsustainable. One factor opening up possibilities for a more sustainable future is the emergence of global consciousness. Given its relative weakness compared to more established forms, like national or local identities, GC cannot grow simply through social learning and imitation, though such forms of cultural continuity have a place. This research found that several established institutions across 6 societies contributed to GC in a significant way. These were education, white collar work (and the higher and more regular income associated with it) and religiosity. Some culturally continuous aspects of industrialized society can contribute to GC. But there is also discontinuity, the most obvious evidence of which is the decrease of GC with increasing age. This suggests a cohort effect, where younger people are learning from their peers and through mass media rather than imitating their elders.

The more innovative (but also more tentative) part of this research revealed that both positive and negative life experiences contributed to the growth of GC over the 3-month longitudinal period measured. Not surprisingly, positive, empathy-building, network-enhancing elements like getting married or welcoming a new infant were the most GC-enhancing events reported. Instrumental gains like receiving a promotion (or getting a better job) also showed positive longitudinal effects.

Less intuitively, death of a close-other enhanced rather than reduced GC. This was not anticipated, given the experimental literature demonstrating that mortality salience [42] causes defensive, ingroup favouring and outgroup derogating thoughts to arise. This research demonstrated the opposite effect: odds of GC increasing after death of a close other were twice that for the individuals who did not experience this. The death of a close-other represents objective discontinuity in people's lives. But it also affords the opportunity to make meaning, and to (re-)create interconnectedness [46]. Maybe death makes people feel small [45], and if the proper rituals are observed to manage such loss, this creates room for new expansive growth. Caring for others (in the form of reporting the serious illness of a close-other) also increased GC. So other ‘negative events’ also appear to have potential to facilitate growth.

There are also some limitations with these data. They were collected during the first year of the COVID-19 pandemic, so this time might have presented unusual societal circumstances for dealing with death or illness. We also have no explanation for why the event of financial loss would have increased GC for this sample, except for circumstances. It is therefore incumbent on future research to provide replication (and extension).

If GC is a cohort effect rather than a maturation effect, then it must be tied to specific forms of learning and experience. In this era, a higher, more regular income affords a wider range of experiences and access to things that may serve to expand horizons. Education can be mind-opening in any era, but especially true in the present one when innovations occur regularly and are documented so they can be institutionally transmitted to a new cohort. These may be features of cultural evolution in the Anthropocene, where all sorts of innovations are regularly recorded and widely disseminated. GC may be particularly adaptive in an era where access to innovation is easy, and the rewards for being open to new people and things generally outweigh the risks of encountering the unfamiliar.

But GC is more than a product of power, plenty, security and industry. It is also a vessel for meaning making, where forming a marriage, adding a new family member, or losing a beloved are used to articulate wider forms of interconnectedness that might be discontinuous with what went before. GC appears to have a fundamentally social component to it that may be a product of evolutionary origins. It appears oriented to not only expanding human social networks. This was a critical element in the rise of our species to planetary dominance, and may become an important element of our shared future where we learn to craft more sustainable narratives about ourselves [57,58].

## Data Availability

The data and code are provided in electronic supplementary material [59].

## Endnotes

1 We further examined the correlations between age and human values, as measured by Gouveia's basic values survey. We found that age had negative associations with promotion values (*r* = −0.304) and excitement values (*r* = −0.235; values of individualism), whereas its correlation with suprapersonal values (roughly equivalent to universalism values in Schwartz’ theory of basic values [53]) was small (*r* =−0.076). By contrast, the components of GC were moderately strongly correlated to suprapersonal values (*r* = 0.4 to 0.6), showing a distinct value profile of GC compared to general prosociality. Details are in electronic supplementary material, table S34.

2 In the main analyses (i.e. LTAs), we exclusively used those participants who responded to both waves (i.e. the stayers), so attrition bias is not a likely explanation for the increases in GC observed after particular life events among those stayers. Therefore, attrition bias is not a threat to the internal validity of this study. However, compared to those who dropped out of the wave 2 survey, those stayers were older, had higher incomes, lower subjective SES and lower religiosity and they were lower on all GC indicators except for respect for cultural diversity (see detailed results in Table 33 of supplementary materials). We do not know for sure if the pattern we observed for the stayers would be applicable to those dropouts. But considering the fact that differences between stayers and dropouts were modest, the threat of attrition bias to external validity is probably limited.

3 Marriage, for example, had a high decay ratio as well as a high advance ratio, suggesting that the character of the marriage (partner) was very important to that event increasing versus decreasing GC; overall however, even subtracting decay ratio from advance ratio, marriage was a positive event for transitioning to higher GC.
